# Protective roles and protective mechanisms of neutralizing antibodies against SARS-CoV-2 infection and their potential clinical implications

**DOI:** 10.3389/fimmu.2023.1055457

**Published:** 2023-01-19

**Authors:** Endeshaw Chekol Abebe, Tadesse Asmamaw Dejenie

**Affiliations:** ^1^ Department of Medical Biochemistry, College of Health Sciences, Debre Tabor University, Debre Tabor, Ethiopia; ^2^ Department of Medical Biochemistry, College of Medicine and Health Sciences, University of Gondar, Gondar, Ethiopia

**Keywords:** neutralizing antibodies, protective role, clinical application, COVID 19 vaccine, SARS-COV-2 variants

## Abstract

Neutralizing antibodies (NAbs) are central players in the humoral immunity that defends the body from SARS-CoV-2 infection by blocking viral entry into host cells and neutralizing their biological effects. Even though NAbs primarily work by neutralizing viral antigens, on some occasions, they may also combat the SARS-CoV-2 virus escaping neutralization by employing several effector mechanisms in collaboration with immune cells like natural killer (NK) cells and phagocytes. Besides their prophylactic and therapeutic roles, antibodies can be used for COVID-19 diagnosis, severity evaluation, and prognosis assessment in clinical practice. Furthermore, the measurement of NAbs could have key implications in determining individual or herd immunity against SARS-CoV-2, vaccine effectiveness, and duration of the humoral protective response, as well as aiding in the selection of suitable individuals who can donate convalescent plasma to treat infected people. Despite all these clinical applications of NAbs, using them in clinical settings can present some challenges. This review discusses the protective functions, possible protective mechanisms against SARS-CoV-2, and potential clinical applications of NAbs in COVID-19. This article also highlights the possible challenges and solutions associated with COVID-19 antibody-based prophylaxis, therapy, and vaccination.

## 1 Introduction

The coronavirus disease of 2019 (COVID-19) is a highly contagious and disastrous disease that has been declared a global pandemic since early 2020 (1). COVID-19, which is caused by severe acute respiratory syndrome coronavirus 2 (SARS-CoV-2), has significantly hit the world with enormous morbidity and mortality while inflicting economic, health, and social impacts since its inception (2, 3). Following SARS-CoV-2 infections, several immune cascades can be triggered that, when effective and balanced, are important to control the pathogenesis of COVID-19 (4, 5). It has been demonstrated that humoral immunity is an essential component of the adaptive immunity that fights against SARS-CoV-2 (6). Antibodies are central players in humoral immune responses produced by the B lymphocytes in response to SARS-CoV-2 infection or vaccinations (7, 8). These antibodies, which recognize and confer protection against foreign objects (antigens), can be non-neutralizing or neutralizing antibodies. Non-neutralizing antibodies (nNAbs), also known as binding or sub-neutralizing antibodies, identify and bind to foreign invaders without interfering with microbial infectivity (9, 10). They do not directly combat pathogens; instead, they alert the body to the presence of viruses so that immune cells can find and kill them. nNAbs exert their effects *via* their conserved crystallizable region (Fc), which interacts with various components of the immune system (11, 12).

Neutralizing antibodies (NAbs) are integral parts of the humoral immune response that defends the body cells from microbial infections, such as SARS-CoV-2 infection, by blocking their entry into a cell and neutralizing their biological action (13). NAbs mediate their function through a variable region of the antibody that binds to the antigen, known as the fragment antigen-binding (Fab) area (11). NAbs, which can be induced by vaccines or prior infection, play a critical role in conferring defense against SARS-CoV-2 infections (14). Clinically, NAbs are currently employed as a key therapeutic component in humans for the treatment of a number of serious pathogenic infections, including SARS-CoV-2 infections (15). The current vaccines, biologic therapy with monoclonal antibodies (mAbs), and convalescent plasma to control SARS-CoV-2 infection are all based on the use of NAbs capable of preventing infection by interfering with the steps of the viral replicative cycle (14, 15). Nonetheless, humoral immunity mediated by NAbs is being challenged by the adverse effects associated with mAbs or COVID-19 vaccines and the advent of new SARS-CoV-2 variants associated with immune evasion. Therefore, a comprehensive understanding of NAb-mediated immune responses to SARS-CoV-2 infection is of paramount importance to guide the development of more potent COVID-19 vaccines and antibody-mediated treatments. This review points out the current knowledge on the role of NAbs in the protection of SARS-CoV-2 infection and their potential clinical implications in COVID-19, as well as summarizes the possible challenges associated with COVID-19 antibody-based prophylaxis, therapy, and vaccinations.

## 2 Protective roles of NAbs in SARS-CoV-2 infection

### 2.1 Protection against primary SARS-CoV-2 infection

Humoral immune responses, most notably those mediated by NAbs, have been amply demonstrated to have a role in host protection against SARS-CoV-2 infections. Several studies confirmed that NAbs are highly predictive of immune protection from SARS-CoV-2 infection (16, 17). Congruently, another report showed that protection against SARS-CoV-2 exposure is positively associated with the development of high neutralizing titers of antibodies. It has been shown that NAbs are correlated with defense against viral infection and provide the best proof that protective immunity is established (18). A plethora of studies has also highlighted that NAbs induced by natural infection or vaccination play a pivotal role in viral clearance during acute SARS-CoV-2 infection (16, 19–23). This was further confirmed by other studies reporting that passive transfer of convalescent animals sera or neutralizing mAbs to naïve animals can have a key defensive role against viral infection (19, 24). Besides their neutralizing role during acute infection, high-affinity SARS-CoV-2 NAbs control disease progression to a more severe type during the chronic phase by facilitating the polarization of the T-cell response towards protective type 1 immunity and preventing the virus-induced pathological type 2 eosinophilic inflammation (25–27).

A growing body of evidence reveals that the protective antibodies produced in response to SARS-CoV-2 infection or vaccination can be immunoglobulin (Ig) G, IgM, and IgA, although IgM and IgA appear in the circulation of many cases of SARS-CoV-2 infection (28, 29). IgG antibodies are considered the main antibodies providing defensive immune responses to fight off the SARS-CoV-2 infection (30). The antibody levels of IgG have been shown to correlate with neutralization and act as excellent predictors of neutralization, though the former has a stronger correlation and is more specific at predicting neutralization (100%), and the latter is a more sensitive predictor of neutralization (98%) (9). When compared to other IgG subclasses, IgG1 and IgG3 have been discovered to have a significant protective role, with IgG3 having up to 50-fold greater neutralizing potency (31). A growing body of research suggests that SARS-CoV-2 infection induces neutralizing IgA that could aid in sero-neutralization as strongly as IgG (32–34). Besides, secretory IgA(sIgA) antibodies in response to SARS-CoV-2 infection are produced and enhance mucosal immunity beneficial against the virus targeting mucosal surfaces (5, 35, 36).

Although it is still a matter of debate on how long the NAbs will last, many different types of literature have reported that anti-SARS-CoV-2 antibodies, which may appear as early as one week after the viral invasion, may persist for several months (37, 38). Consistent findings have also been reported by a recent meta-analysis demonstrating that NAbs can be detected 7-15 days after symptom onset in individuals with naturally acquired SARS-CoV-2 infection, while their neutralizing activity gradually declines between 1.3 month to 6.2 months (39, 40). Particularly, anti-SARS-CoV-2 S-specific IgM antibodies become detectable after the fourth day of the infection, peak during the first week of disease onset, and persist for 20 days to a month before gradually declining (41, 42). Plasma IgGs, on the other hand, are detected within the first two weeks of symptom onset, but reach their maximum quantities between the 16^th^ and 50th day, and then decline thereafter (42–44). But it has been demonstrated that IgG can persist in the blood for several months and can still be seen at least a year after infection (45–47). Neutralizing IgA antibodies against SARS-CoV-2 can be detected as early as a week after symptoms appear, they peak between the 15^th^ and 30^th^ day, and disappear at 45–50 days later (32, 44, 48).

### 2.2 Protection against SARS-CoV-2 reinfection

In addition to their defensive function against primary infection, antibody-mediated immunity acquired through natural infection or vaccination can offer some protection against reinfection and/or lower the risk of clinically significant outcomes (23, 49–51). Nonetheless, whether an individual is protected from SARS-CoV-2 infection after recovery depends on the quantity and quality of the antibodies produced. Antibodies of adequate quality (neutralizing) and sufficient quantity in a person recovering from SARS-CoV-2 infection are protective against re-infection (49). A strong correlation exists between the level of neutralization and immunological defense against SARS-CoV-2, with higher levels indicating higher-quality antibodies and protection (17). The presence of adequate antibodies in response to prior infection is also strongly associated with resistance to reinfection, whereas insufficient humoral defenses cause reinfection (23).

Several investigations involving non-human primate models challenged with SARS-CoV-2 developed potent antibody responses and are protected from reinfection by the same strains for at least some time (50, 51). Notably, an observational study showed that the majority of patients with COVID-19 seroconversion potentially provide immunity to reinfection (38). Consistently, individuals who have recovered from mild COVID-19 develop reasonably robust antibody responses to S protein, which correlate significantly with neutralization of re-infection with SARS-CoV-2 (38). It has also been demonstrated that seropositive recovered subjects have 89% protection from reinfection. Furthermore, recent publications reported that people infected with wild-type SARS-CoV-2 may produce potent human broadly SARS-CoV-2–neutralizing IgA and IgG antibodies effective against Omicron BA.1 and BA.2 (5, 17).

Nevertheless, it is not clearly known how long protective immunity against prior infection will last and confer protection combating re-infection. Studies have reported that SARS-CoV-2 antibody responses may persist for several months after infection and provide protection against the second bout of infection (52). But the primary immune responses show an ineluctably gradual waning in convalescent patients, with a possible risk of re-infection (17, 53). In agreement, another report showed low antibody titers or quick fading of antibodies against SARS-CoV-2 in recovering patients (48). Zhang et al. also demonstrated that reinfections may occur during the convalescent stage in patients who had SARS-CoV-2 infection-induced humoral immunity (54). Notably, suboptimal antibody-mediated immune responses to prior infection or vaccination that may increase the risk of reinfection have been prominently observed in those older or immunocompromised individuals (55). Collectively, NAb levels have been shown to decline from 2-3 months after initial infection in some reports, while significantly falling after 6-8 months after the onset of symptoms in other studies (56–58). This reveals the possibility of reinfection with a second bout of SARS-CoV-2 infection, even shortly after recovery from the primary infection.

Recent real-world data from vaccine-rollout programs have shown the protective cut-off titer of antibodies induced by COVID-19 vaccines or infection (**Table 1**). Both nNAbs and NAbs have been demonstrated to be potential correlates of protection (CoP) against SARS-CoV-2 infection, although diverse lines of evidence have established postvaccination NAb titers as a better CoP, which is an immunological metric used to assess vaccine efficacy against SARS-CoV-2 infection (63, 66). Vaccine efficacy is the percentage reduction in the average risk of COVID-19 among vaccine recipients as opposed to the risk among placebo receivers (66).

**Table 1:** A summary table on the protective antibody titer and vaccine efficacy against SARS-CoV-2 infections.

Reference	Study type	COVID-19 vaccine	Findings
Gilbert et al. (59).	COVE clinical trial	mRNA-1273 vaccine	• Vaccine recipients with day 57 postvaccination 50% neutralization titers (ID50) 10, 100, and 1000 IU50/ml have estimated vaccine efficacies of 78%, 91%, and 96%, respectively, reducing COVID-19-risk by 5.5-fold
Fong et al. (60).	ENSEMBLE trial	Ad26.COV2.S vaccine	• The estimated vaccine efficacy was 60% at non-quantifiable D29 ID50 (<2.7 IU50/ml). • The vaccine efficacy increased to 78% at 9.9 IU50/ml and to 89% at 96.3 IU50/ml.
Fong et al. (61).	PREVENT-19 trial	NVX-CoV2373 vaccine	• The NVX-CoV2373 vaccine efficacy to reduce the risk of acquiring COVID-19 at 59 days at NAb ID50 titers of 50, 100, 1000, and 7230 IU50/ml were 75.7%, 81.7%, 92.8%, and 96.8%, respectively.
Ramasamy et al. (62).	COV002 trial	ChAdOx1 nCoV-19 vaccine	• Median anti-S IgG responses 28 days after the booster dose were 20713AU/ml for 18-55 years, 16 170 AU/mL for 56-69 years, and 17561 AU/mL for ≥70 years. • NAb titers after a boost dose at day 42 were standard-dose groups: 193 microneutralization assay (MNA_80_) for 18-55 years; 144 for 56-69 years, and 161 for ≥70 years.
Feng et al. (63).	COV002 trial	ChAdOx1 nCoV-19 vaccine	• A vaccine efficacy of 80% against symptomatic SARS-CoV-2 infection using this vaccine was achieved with 264BAU/ml for anti-S antibodies, 506 BAU/ml for anti-RBD antibodies, 26IU/ml (or 185 ID50) for pseudovirus NAb titer, and 247 NF50 for live-virus neutralization.
Li et al. (64).	COV006 trial	ChAdOx1 nCoV-19 vaccine,	• At day 28 after the first dose of vaccine, anti-S IgG were 24816AU/mL in 12–17 years and 32709 AU/mL in 6–11 years. • These values were increased after the second dose of the vaccine at 112-day. • Pseudovirus SARS-CoV-2 NAb titers also peaked at day 28 after the second dose, which is higher in those 6–11 years age (885 IC50) than in those aged 12–17 years (299 IC50) in the 112-day interval group.
Christiano et al. (65).	Observational study	Vaccines and SARS-COV-2 infection	• The median NAb levels for COVID-19 patients without symptoms were 281.3 BAU/ml. • The NAb levels of 769.4 BAU/ml for symptomatic patient; 351.65 BAU/ml for the vaccinated cohort and 983 BAU/ml for those vaccinated with second dose. • NAb levels of 408.6 BAU/ml was established as cut-off of to identify subjects with a low risk of infection.

AU, arbitrary units; BAU, binding antibody units; IC50=half-maximal inhibitory concentration; ID50, 50% inhibitory dilution; IU, international unit; NAbs, neutralizing antibodies; NF50, normalized neutralization titers.

## 3 Protective mechanisms of NAbs against SARS-CoV-2 infection

**Figure 1:**
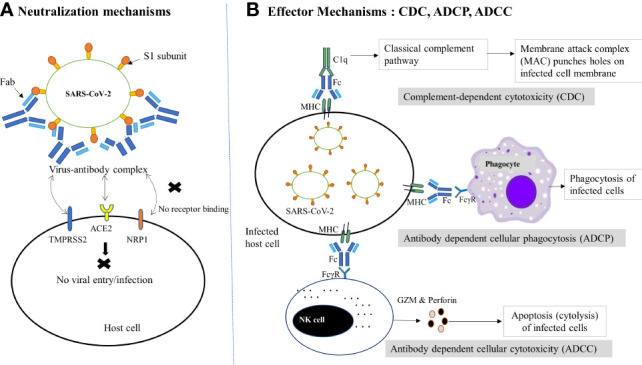
Schematic diagram of NAb-mediated protective mechanisms against SARS-CoV-2 infection. **(A)** Neutralization mechanisms: Following the binding of NAbs (via Fab) to the RBD of the S1 subunit of SARS-CoV-2, the virus-antibody complex is formed, which prevents the viral binding to host receptor (ACE2) or coreceptors (TMPRSS2, NRP1 etc.) or changes the conformation of the S protein to block viral entry into host cells, thereby preventing subsequent membrane fusion or infection. **(B)** Effector mechanisms: The binding of antibody-bound infected host cells (via MHC and Fc) to the FcγR of the immune cells and complements (C1q) trigger several effector mechanisms to kill the virus infected cells. In the CDC pathway, complement activates the classical complement pathway and induces cell death by the formation of MAC. On the other hand, phagocytes interact with MHC and result in phagocytosis of infected cells *via* ADCP. Similarly, the interaction of FcγR-expressing NK cells with the Fc of the antibody complex leads to cytotoxic granule release (GZM and perforin) and hence cytolysis (ADCC). ACE2, Angiotensin-converting enzyme 2; ADCC, Antibody-dependent cellular cytotoxicity; ADCP, Antibody-dependent cellular phagocytosis; CDC, complement-dependent cytotoxicity; Fab, fragment antigen-binding region; Fc, crystallizable fragment; FcγR, Fc gamma receptor; GZM, Granzyme; MAC, Membrane attack complex; MHC, major histocompatibility complex; NAbs, Neutralizing antibodies; NK, Natural killer cell; NRP1, neuropilin 1;TMPRSS2, transmembrane serine protease 2.

NAbs employ a range of proposed defensive mechanisms to eradicate SARS-CoV-2, including direct inhibition of viral entry to host cells (neutralization) and post-binding inhibition (effector functions) (**Figure 1**) (67). Neutralization, which typically blocks the early steps of the viral replication cycle, is the primary defensive mechanism of NAbs to prevent SARS-CoV-2 (68, 69). It is achieved by the binding of antibodies through their Fab region to viral surface proteins, blocking their interaction with the host cell receptor and disabling the viral infectivity without the involvement of any other antibody or immune cell activity (15, 70). This process of losing the virus’s ability to replicate and cause severe infection and rendering the infectious agent no longer infectious or pathogenic to cause disease is known as neutralization. However, the types of targeted viral proteins have been found to influence the production of potent antibodies that are potentially neutralizing SARS-CoV-2 infection. Although anti-SARS-CoV-2 host antibodies may target both the structural and non-structural proteins, two structural proteins, the nucleoprotein (N) and spike (S) protein, have been discovered to be highly immunogenic viral antigens recognized by antibodies (20, 37, 38).

S protein is a large trimeric glycoprotein integrated over the viral surface and possesses S1 and S2 subunits. The S subunit contains a 223 amino acid long immunologically important domain found near its C-terminus called the receptor-binding domain (RBD). During host cell infections, the SARS-CoV-2 uses RBD to bind to the host cell surface receptors, the angiotensin-converting enzyme 2 (ACE2) receptor, and mediate subsequent membrane fusion (20, 71–74). The disruption of this RBD–ACE2 interaction prevents SARS-CoV-2 entry into host cells, making RBD a prime target to induce robust antibody-mediated immune responses that are potentially neutralizing and protective of SARS-CoV-2 infection (38, 71, 72). On the other hand, N protein is a structural protein that is found inside the SARS-CoV-2 virus or infected cells and involved in RNA transcription, replication, and packaging of the encapsidated genome into virions. Although N protein is shown to be a highly immunogenic viral protein abundantly expressed during infections, the antibodies produced against this protein are unlikely to directly neutralize SARS-CoV-2. This might be due to the biological function and hidden location of N protein, which induce little or no NAb (38, 75, 76). In comparison, patients with antibodies against the N protein have a weaker neutralizing potential than those with antibodies against the S1-RBD. Besides, the antibodies that bind N proteins do not correlate with those that bind S1-RBD or have neutralizing properties, revealing that, unlike anti-S1-RBD antibodies, anti-N protein antibodies failed to elicit NAb production (77, 78). Moreover, compared to anti-S antibodies, anti-N antibodies showed lower persistence following SARS-CoV-2 infection for up to a year (58).

In addition, NAb binding to the viral protein epitopes that interact with host cell coreceptors is another suggested protective mechanism against viral infection (75). Although many NAbs can block the attachment of the RBD with ACE2, there is the possibility that several NAbs bind to the non-RBD segment of the S protein or the RBD region that do not compete with the ACE2 receptor but recognize epitopes other than the ACE2-binding motif (9, 71, 79). A number of host cell coreceptors or alternative receptors that may be involved in SARS-CoV-2 infectivity and targeted by NAbs include glucose-regulating protein 78 (GRP78), dipeptidyl peptidase 4 (DPP4), AXL, CD147, neuropilin 1(NRP1), TM serine protease 2 (TMPRSS2), lectins, and vimentin (80–86). Therefore, NAb-mediated interruption of the host coreceptor-viral protein interactions could be an alternative mechanism by which antibodies defend the host cells from viral infection. Besides viral entry blocking, NAbs have been found effective as virus-neutralizers by modifying the conformation of the S protein and blocking membrane fusion. NAbs dock to viral epitopes that are not essential for host cell receptor binding but are necessary for conformational changes needed for virus-host cell membrane fusion required for the virus to reach the cytoplasm of host cells (72). Moreover, binding of NAbs to viral proteins necessary for host cell receptor attachment, specifically distal epitopes or internalized portion of fusogenic proteins, to block complete membrane fusion is another way antibodies shield host cells from viral infection. This mechanism of neutralization can occur once the virus is inside endosomes; the antibodies’ interaction with viral surface proteins prevents the modifications required for the viral membrane fusion, which prevents viral infection (11).

In some instances, when viruses escape neutralization and infect host cells, the elimination of infected cells could be accomplished through a variety of antibody-mediated effector mechanisms, involving antibody-dependent cell cytotoxicity(ADCC), antibody-dependent cellular phagocytosis (ADCP), and antibody-mediated complement-dependent cytotoxicity (CDC) (67, 87). NAbs employ these non-neutralizing effector mechanisms by directly docking through their Fc area to the specific Fcγ receptors (FcγR) expressed on the surface of many immune cells like natural killer (NK) cells, macrophages, and neutrophils. Once these human immune cells have bound to the FcγR of antibodies, such as FcγRI, IIA, IIC, IIIA, and IIIB, they get activated in the battle against infectious agents, leading to ADCC, ADCP, and CDC by NK cells, phagocytes, and complement systems, respectively (9, 79). In these effector mechanisms, nNAbs may work alongside NAbs to signal the immune cells to harmful invaders that need to be eliminated. Besides, nNAbs can also exert these Fc-dependent antiviral effector functions, although they bind the viruses differently and hence are unable to neutralize them effectively and necessarily decrease the infection (88–90). Cumulatively, despite the fact that nNAbs are beneficial, NAbs are more effective at battling infections, including SARS-COV-2.

## 4 Clinical implications of antibodies in COVID-19

In addition to their biological role, antibodies have been observed to play a crucial role in clinical settings, including as markers for COVID-19 diagnosis, determining the immune status and duration of post-COVID-19 infection/vaccination, and serving as disease severity, and prognosis markers. Besides, antibodies have essential implications in designing COVID-19 therapy, prophylaxis, and vaccines, as discussed in this part of the review.

### 4.1 Diagnostic value of antibodies in COVID-19

In the current COVID-19 pandemic situation, different diagnostic modalities are being used to detect whether an individual is infected with SARS-CoV-2 or not. A number of serological assays have already been approved for clinical practice to detect antibodies against SARS-CoV-2, identify individuals infected with the virus, and thus diagnose COVID-19 infection (9, 38). In serological tests, antibodies against the highly immunogenic N and S viral proteins are frequently used. But due to a conserved N protein across the various coronaviruses (including non-SARS-CoV-2), N protein-based antibody assays have a greater false-positive rate (less specificity) and lesser performance than anti-S antibodies in differentiating COVID-19 patients from controls (6, 20, 38).

Because anti-SARS-CoV-2 antibodies can be found in the blood as early as 10 days after symptom onset, and because COVID-19 vaccination is now widely used, serological tests are nowadays rarely used to diagnose acute infections on their own. Instead, those tests using anti-SARS-CoV-2 antibodies could be potential diagnostic tools to complement a real-time polymerase chain reaction (RT-PCR)-based diagnosis to enhance the detection window of SARS-CoV-2 infection and reduce false-negative RT-PCR testing (20). Moreover, with the emergence of SARS-CoV-2 variants, concerns have also been raised over the ability of serological assays to detect antibodies to the new variants. The Food and Drug Administration (FDA) also cautions against using antibody-based tests to identify an active infection because they only identify the antibodies that the immune system produces in response to a virus, not the virus itself (91).

### 4.2 Antibody’s role in determining the levels and duration of protective immunity

Antibodies could have an essential utility for the evaluation of protective individual or herd immunity produced against SARS-CoV-2 infection or vaccination (67). In response to SARS-CoV-2 infections or vaccinations, a person may generate antibodies that may offer protective immunity to fend against re/infection. The presence of antibodies does not necessarily indicate immunity against potential re-infection; rather, neutralization titer is highly predictive of immune protection, providing evidence on SARS-CoV-2 immune protection and vaccine efficacy (17). Moreover, the evaluation of NAb levels is useful for the determination of the longevity of the protective humoral response and supporting donor selection criteria for convalescent plasma therapy (67). This reveals that measuring NAb titer is crucial to ascertain whether antibodies are virus-neutralizing and aid in viral clearance and whether they can confer long-lasting protective immunity.

A variety of serology assays involving anti-N or anti-S antibodies can be employed for measuring the humoral response to SARS-CoV-2, including antibody levels, subclasses, avidity, and neutralization activity (92–94). Anti-N serology tests can be used in settings where prior infection with SARS-CoV-2 is suspected. However, serology tests using anti-N protein portray not just the presence of antibodies from prior exposure to the SARS-CoV-2 but also to other related viruses; hence, they may not be a reliable indicator of the presence of NAbs. Thus, using anti-N protein-based serology tests to evaluate long-term COVID-19 immunity may potentially be misleading. Contrarily, anti-S serology assays, especially those involving the anti-S1 subunit and anti-RBD antibodies, detect antibodies produced after prior SARS-CoV-2 infection and vaccination and thus better indicate the neutralizing capacity of antibodies (78, 95).

The antibody-based detection methods can be qualitative, semi-quantitative, or quantitative (96). Qualitative serological tests reveal the existence of antibodies that may help determine whether the patient has developed a humoral immune response against SARS-CoV-2. This type of test measures the host immune response in the form of IgM, IgA, or IgG following infection or vaccination. However, qualitative serologic tests do not provide information on the quantity of antibodies present in a patient. Hence, this test should not be used to assess the immune status of individuals post-COVID-19 infection/vaccination (97). Whereas, semi-quantitative serological assays quantify estimated antibody concentrations rather than their precise values and report the results using arbitrary units. Despite the fact that semi-quantitative assays aid in the diagnosis of COVID-19 and the determination of antibody and neutralization titers, their clinical applicability in the study of the immune status of SARS-CoV-2 infected or vaccinated people remains theoretical due to the test’s semi-quantitative nature (96). Quantitative serologic tests are the type of assays that are lately developed to precisely measure antibody levels in COVID-19 patients. Remarkably, high-throughput quantitative serological tests using the S1 subunit and RBD are becoming increasingly important to determine antibody levels, neutralization titers, and neutralization potency index (97, 98).

Although numerous serological tests are developed to evaluate the binding antibodies, there are only a few neutralizations assays that measure the functional ability of NAbs to block SARS-CoV-2 entry into host cells. The reference test for assessing the ability of antibodies to prevent the virus from entering host cells is the virus neutralization test (VNT) (99). In particular, plaque reduction neutralization tests (PRNT) or conventional neutralization tests (cVNT) are considered the gold standard test for evaluating antibody neutralization capacity against SARS-CoV-2 (100, 101). But this test is a labor-intensive assay that demands a high degree of expertise and exposes laboratory professionals to infection risks as live virus and cell culture are involved. Besides, PRNT has very a low throughput, a long turnaround time, and is difficult to automate. Due to these limitations, it is unsuitable and less reproducible for use in large-scale screening programs (24, 102). Consequently, VNTs are nowadays being replaced by a number of other cost-effective and feasible serological assays, which are frequently used as part of research and diagnostics to identify NAbs and determine potential protective antibody titers after infection or vaccination.

Currently, several serological assays have been approved for clinical practice by the FDA Emergency Use Authorization (EUA) (**Table 2**). Collectively, they are instrumental for long-term monitoring of humoral immune response to infection or vaccines, for measuring antibody-longevity and waning, determining threshold values that can correlate to protection, quantifying seroprevalence in the community to estimate herd immunity, and determining the fatality rate of infection. Besides, serological tests are crucial for recruiting suitable individuals for clinical trials of vaccine or therapy development, and for evaluating the efficacy of different vaccine candidates. and for collecting plasma donations from convalescent COVID-19 patients with the highest levels of antibodies (9, 111, 112).

**Table 2:** Some EUA authorized serological assays measuring humoral immune response or antibody levels.

Serology test	Developer	Technology	Purpose	Test performance	Refer
EUROIMMUN Anti-SARS-CoV-2-NCP ELISA (IgG)	EUROIMMUN US, Inc.	Semi-quantitative ELISA	Measures antibodies binding to modified N protein	80%-94.8% sensitivity, 99.8% specificity	(91, 103).
EUROIMMUN Anti-SARS-CoV-2 S1 Curve ELISA (IgG, IgA)	EUROIMMUN US, Inc.	Semi-quantitative ELISA	Detect antibodies binding to the S1 subunit of the S protein	91.1% sensitivity, 100% specificity	(91, 104, 105).
Abbott Architect SARS-CoV-2 IgG assay	Abbott	High Throughput CMIA	Designed to measure IgG antibodies binding the N protein	100% sensitivity, 99.6|% specificity	(91, 106).
Abbott AdviseDx SARS-CoV-2 IgG II assays	Abbott Laboratories Inc.	Semi-quantitative High Throughput CMIA	Detect neutralization titer of antibodies by employing S protein	98.1% sensitivity and 99.6% specificity	(55, 91).
Roche Elecsys anti-SARS-CoV-2 assay	Roche	High throughput ECLIA	Measures the total anti-N antibody titers	100% sensitivity and 99.8% specificity	(55, 91).
Roche Elecsys anti-SARS-CoV-2 S	Roche Diagnostics International Ltd	Semi-quantitative high throughput ECLIA	Detect the level and duration of immune response by using the RBD of the S protein	96.6% sensitivity and 100% specificity	(55, 91).
cPass SARS-CoV-2 NAb Detection Kit	GenScript USA Inc.	ELISA	Measure the NAb-mediated blockage of the RBD-ACE2 interactions in an isotype- and species-independent manner	100% sensitivity and 100% specificity	(91, 107).
LumiraDx UK Ltd. LumiraDx SARS-CoV-2 Ab Test	LumiraDx UK Ltd.	Fluorescence Immunoassay	Evaluate humoral immune response using S protein	100% sensitivity and 98.8% specificity	(91, 108).
Phadia AB EliA SARS-CoV-2-Sp1 IgG Test	Phadia AB	Semi-quantitative Fluoroenzyme Immunoassay	Determine anti-S protein antibodies	97.6% sensitivity and 99.4% specificity	(91, 109).
LIAISON^®^ SARS-CoV-2 TrimericS IgG assay	DiaSorin, Inc.	semi-quantitative High Throughput CLIA	measure IgG antibodies to the trimeric S protein of the SARS-CoV-2	99.7% sensitivity and 99.5% specificity	(91, 110).

CIMA, chemiluminescent microparticle immunoassay; CLIA, chemiluminescent immune-assay; ECLIA, Electro-chemiluminescent immune-assay; ELISA, enzyme-linked immunosorbent assay; EUA, emergency use authorization; Ig, immunoglobulin; NAbs, neutralizing antibodies, NCP, nucleocapsid.

### 4.3 Antibodies as severity and prognostic markers of COVID-19

Accumulated evidence revealed that antibodies may be used as severity and prognostic markers in COVID-19. A study by Garcia-Beltran and his coworkers demonstrated that antibody levels, neutralization titer, and neutralization potency are associated with disease severity and may predict patient clinical outcomes (survival) (9). Higher antibody titers have been found to have a significant association with severe disease and worse outcomes (9). Yamayoshi et al. also found higher antibody titers in patients with severe infections than in those with mild or moderate infections (113). Consistently, several other studies have shown that more severe COVID-19 cases are associated with higher anti-RBD and anti-S antibody levels (114, 115). In line with this, another study found that immune activation and high antibody production from extrafollicular B cells play a pathogenic role in severely ill patients (116). This could be due to antibody overproduction that results in hyperactive inflammatory responses and uncontrolled viral replication *via* FcR-or complement-dependent effector mechanisms known as antibody-dependent enhancement (ADE). Moreover, many publications have reported that neutralization titers, in addition to antibody levels, can dictate the clinical outcomes of COVID-19 patients, with higher neutralizing titers being linked with worse patient outcomes (114, 115, 117–119). The correlation of higher antibody and neutralization titers with more intense COVID-19 symptoms suggests that robust antibody responses may not be sufficient enough to fend off serious illnesses. Antibody and neutralization titers, on the other hand, may have clinical relevance as severity and prognostic markers in COVID-infected patients.

Additionally, Garcia-Beltran et al. showed that the neutralization potency index may serve as a better severity and prognostic marker to assess disease severity and patient outcomes in COVID-19 (9). Quantification of neutralization potency is used to assess the quality of anti-RBD IgG antibodies regardless of their amount. Intriguingly, the neutralization potency of anti-RBD antibodies has been found to be a strong indicator of patient outcomes or survival in which patients with more potent virus-neutralizing antibodies appear to have better clinical outcomes or are more likely to survive. Further analysis of this study demonstrated that an index of 100 or above predicts 100% 30-day survival, whereas an index of less than 100 predicts 87% 30-day survival (9). Besides, the neutralization potency index can be used to predict disease severity in that patients with more severe or fatal COVID-19 appear to have substantially less effective NAbs than those with milder or asymptomatic cases (9). The worsening of disease severity is postulated to be caused by high levels of antibodies and sub-optimal neutralization potency that leads to excessive inflammatory responses and viral over-replication *via* ADE (20). Altogether, measuring a patient’s anti-RBD antibody neutralization potency index is a promising biomarker of severity that could be employed as a valuable tool for physicians seeking COVID-19 patient risk assessment to identify patients at risk of severe disease or death and guide treatment options.

### 4.4 Potential therapeutic and prophylactic roles of antibodies in COVID-19

Multiple studies have shown that passive antibody transfer has important implications for conferring a state of protective immunity in prophylactic or therapeutic settings (120–123). This involves the passive transfusion of recombinantly produced antibodies or antibodies from the plasma of convalescent patients to a non-immune person to provide immediate protection against severe SARS-CoV-2 infection (111, 124). In the absence of effective antiviral therapeutics, convalescent sera therapy has been used to treat patients with the severe disease during many viral disease outbreaks (125). Prior studies have demonstrated that plasma NAbs from recovered patients were successfully employed in the passive antibody therapy for patients infected with SARS-CoV, influenza virus A, and Ebola virus (76, 126–130). Similarly, because severe COVID-19 urges rapid intervention during a pandemic, sera from recovered patients have been studied for the discovery of possibly effective therapy against SARS-CoV-2 for severe patients (20).

Accumulating data shows that the use of recovered sera from COVID-19 patients to treat critically ill patients has been observed to be successful with minimal or no adverse effects (131–134). This agrees with another study indicating a higher discharge rate and no adverse effects among patients treated with convalescent sera earlier in infection (131). Furthermore, several prior small and large-scale studies on severe COVID-19 patients have shown that convalescent sera, which are safe and potently neutralizing, may improve patient outcomes in the absence of effective antiviral therapeutics (9, 16, 131–138). According to a Chinese pilot investigation by Chen et al, transfusion of convalescent sera with NAb titers above 1:640 (in 200ml of plasma) is well tolerated and may improve patient outcomes through neutralizing viremia in severe COVID-19 cases (123). Whereas a case report from Indonesia recommends that the titer of SARS-CoV-2 NAb should be greater than 1:320 to effectively treat COVID-19 patients (120). Another recent randomized clinical trial also revealed that the use of high-titer convalescent plasma is beneficial in immunocompromised patients who have not yet had an antibody response (122). Convalescent plasma immunotherapy has been demonstrated to result in a longer survival time and a shorter hospital stay, especially when it is given early to COVID-19 patients (120). FDA has authorized convalescent plasma for emergency use in the treatment of hospitalized COVID-19 patients (139).

On the contrary, a large clinical study conducted by Sullivan et al. reported that patients who received convalescent plasma transfusions and control groups had an insignificant difference in the rate of COVID-19-related hospitalization within 28 days, revealing the minimal benefit of convalescent plasma (140). Similarly, high-titer convalescent plasma that is collected from donors after natural infection does not have a benefit in unselected patients who visit the emergency room or are admitted to the hospital (141). Another recent randomized clinical trial also reports that convalescent plasma does not improve survival or decrease the requirement for mechanical ventilation while having large expenses (142). Taken together, the plasma of recovered patients has less benefit in critically ill patients or non-severe COVID-19 patients.

Passive immunotherapy using engineered anti-SARS-CoV-2 mAbs that target the S protein has been shown to have clinical benefits in treating patients with COVID-19 (143, 144). SARS-CoV-2-infected mice receiving mAbs against the S protein were observed to reduce viral titers and lung pathology, suggesting the protective effect of antibodies against SARS-CoV-2 (19). Recently, the mAbs engineered from NAbs isolated initially from convalescent COVID-19 patients are advancing into antiviral treatment. It has been shown that mAb therapy more effectively improves patient outcomes when given to outpatients early in the course of COVID-19 or to those without an antibody response (145, 146). To date, although there is no currently available mAb product for post-exposure prophylaxis of SARS-CoV-2 infection, a few mAbs have received EUAs from the FDA for the treatment of selected hospitalized COVID-19 patients. Bebtelovimab, tixagevimab plus cilgavimab, bamlanivimab plus etesevimab, casirivimab plus imdevimab, and sotrovimab are the current approved mAbs for COVID-19 therapy (147).

However, the anticipated activity of different anti-SARS-CoV-2 mAb therapies varies significantly depending on the circulating variant. Presently, because of the high global prevalence of the omicron variant, bamlanivimab plus etesevimab, casirivimab plus imdevimab, and sotrovimab have been reported to provide little clinical benefit for patients with COVID-19 caused by the omicron variant (147, 148). This suggests the need for more effective mAb therapies against SARS-CoV-2, including emerging variants. A recent report indicates that IgM or IgA mAbs, which possess different Fab domains to interact with multiple antigen-binding sites, have been shown to have a dramatically higher efficiency of neutralization against SARS-CoV-2 than the currently approved IgG mAbs with the same Fab domain (149). This exciting finding highlights that IgM and IgA mAbs could be used as effective and affordable therapeutic options for COVID-19, including those caused by new variants, although further supporting evidence is required.

### 4.5 Roles of antibodies in vaccine development

Since the outbreak of COVID-19, scientists have been working tirelessly to develop an effective vaccine against SARS-CoV-2. COVID-19 vaccine development is currently advancing at an unheard-of rate, with numerous vaccines in the early phases of preclinical and clinical studies, some of which have already received approval for use. These COVID-19 vaccines can be mRNA vaccines, viral vector vaccines, protein-based vaccines, or attenuated coronavirus vaccines that induce protective NAbs (150–155). The mRNA vaccines are designed from mRNA that forms genetic instructions for producing the S protein of SARS-CoV-2. The S protein then gets released into the body and elicits humoral immune responses (or NAbs). The mRNA-1273 (from Moderna) and BNT162b2 (from Pfizer-BioNTech) vaccines are lipid nanoparticle encapsulated mRNA vaccines that encode the full-length S protein of SARS-CoV-2 (156, 157). The mRNA-1273 vaccine is recommended for those aged 6 months or older, with two intramuscular doses given at 4-8 weeks’ interval, depending on their age. Similarly, the BNT162b2 vaccine is authorized for those aged 6 months and older using two or three doses, with an adjustment in the recommended dosage for those between 6 months to 11 years. Full vaccination with these two vaccines is shown to have an efficacy above 95% in preventing COVID-19. But the booster doses are required after 4-6 months of completion of the primary series of these vaccines using the same or another COVID-19 vaccine (150, 152).

In addition to mRNA vaccines, COVID-19 vaccines can be viral vector vaccines, including the AZD1222 from Oxford-AstraZeneca, the JNJ-78436735 from Johnson and Johnson, and the Sputnik V from the Gamaleya Research Institute.COVID-19 viral vector vaccines are made from modified versions of various harmless viral vectors, such as adenoviruses, to deliver important instructions to host cells. After getting into a human cell, these vaccines will use the cell’s machinery to express a harmless piece of the virus or the S protein and trigger the immune system to produce NAbs and stimulate other immune cells to fight off what it thinks is an infection (151, 153, 158–161). Specifically, the AstraZeneca (AZD-1222) vaccine is a non-replicative chimpanzee adenoviral vector ChAdOx1 containing S protein (159). As per recent recommendations, it is administered as a 2-dose intramuscular injection spaced 8 to 12 weeks apart and has achieved nearly 79% efficacy against COVID-19 (151, 158, 159). On the other hand, the JNJ-78436735 (Ad26.COV2.S) vaccine is designed using adenovirus 26 (Ad26) and administered as a single intramuscular shot to provide 66-9%-76.7% of protective efficacy after two weeks of vaccination (162). The Sputnik V vaccine is also a vector vaccine using Ad26 and Ad5 as vectors for the expression of the S protein of SARS-CoV-2. This vaccine is given at two different times to achieve 91.6% efficacy and is now authorized for emergency use in many other countries (160, 161).

Furthermore, several COVID-19 vaccines contain coronavirus whole proteins or fragments of them with no genetic material, which can be with or without nanoparticle packaging, known as protein-based COVID-19 vaccines. The adjuvanted NVX-CoV2373 vaccine from Novavax is a protein-based vaccine that incorporates the S protein of SARS-CoV-2 as well as matrix (M) adjuvant that enhances the immune response of the vaccinated people (154). Currently, the FDA has granted an expanded EUA for the adjuvanted Novavax (NVX-CoV2373) vaccine to be administered as a two-dose primary series (with an 8-week interval) for active immunization against COVID-19 in people aged 12 and older (163). Evidence from different phases of the clinical trials indicates that the efficacy of the Novavax vaccine against mild, moderate, and severe diseases is about 90% (164). However, the NAbs produced against this vaccine cannot last longer than 6 months, and hence, a booster dose is generally recommended after 4-6 months of the completion of primary vaccination with an authorized COVID-19 vaccine (165). Moreover, COVID-19 vaccines can also be created from weakened, inactivated, or chemically killed coronaviruses to produce NAbs against the viruses in vaccinated people. Sinovac, BBIBP-CorV, sinopharm-Wuhan, and Bharar Biotech are some examples of attenuated COVID-19 vaccines. CoronaVac (Sinovac) is produced by Sinovac Biotech and given in two intramuscular injections with 14 days gap (166). Whereas, BBIBP-CorV is produced by a Chinese company (Sinopharm) and administered intramuscularly in two doses separated by 21 days to bring nearly 79.3% efficacy. This vaccine is currently authorized for emergency use in several countries (166).

Overall, COVID-19 vaccines are thought to produce a neutralizing type of antibodies and promote T-cell response skewing towards Th1 polarization and blocking Th2 response against COVID-19 (111). NAbs have been shown to be the main correlate of protection for COVID-19 vaccines (59, 167, 168). An increasing body of evidence demonstrates that vaccines against SARS-CoV-2 have been shown to elicit neutralization titer comparable to those observed in naturally infected persons (75, 169). Moreover, a number of successful COVID-19 vaccines with a protection rate greater than 90%, such as BNT162b2, Moderna, and NVX-CoV2373 vaccines, were reported to exhibit Th1-cell-skewed responses of their S protein antigens during preclinical and clinical studies (170). However, while a plethora of studies have demonstrated good humoral responses against SARS-CoV-2 after a few days of vaccination, they do not last long and gradually wane (171). This is evident from a considerable loss of protection or neutralization levels measured from the post-immunization neutralization titer, highlighting the need for booster immunization (167). Currently, WHO recommends at least one booster dose after completion of the primary cycle of many of the COVID-19 vaccines in individuals immunized a few months before.

## 5 Challenges of antibody-based COVID-19 prophylaxis, therapy, and vaccination

### 5.1 Challenges associated with convalescent plasma and mAbs and possible solutions

Available evidence indicates that the prophylactic or therapeutic use of convalescent plasma may trigger the production of antibodies that could then facilitate immunopathology such as type 2 inflammatory response, leading to more serious illness or death (111, 172). This adverse condition may happen when a large number of antibodies with less potent neutralizing ability is administered and result in the deterioration of the disease condition or death. Similarly, another report observed ADE with sera from COVID-19 convalescents in an FcR- and ACE2-dependent manner (173). The FDA-approved mAb therapies, such as casirivimab, imdevimab, and sotrovimab, have been reported to cause ADE in FcR- and ACE2-positive cells (174). Several recent studies have also shown that anti-S-protein mAbs can function as ADE-causing antibodies (175, 176). This raises a serious concern over the use of convalescent plasma or mAbs as a treatment or prophylactic strategy. Thus, measuring the neutralization titers and potency of the patient’s antibodies to determine the efficacy and safety of convalescent plasma is required before the transfusion of the convalescent plasma to reduce the risk of potential adverse reactions. Besides, the convalescent plasma of those patients with comorbidities and those who have a high risk of developing immunopathology should be excluded from transfusion (79).

### 5.2 Challenges associated with COVID-19 vaccines and possible solutions

Despite the prophylactic advantage, COVID-19 vaccines have some downsides, as demonstrated by several publications. A number of reports showed that the emergence of ADE is a potential clinical safety risk associated with COVID-19 vaccines (173, 176, 177). COVID-19 vaccines that contain non-neutralizing antigen targets are proposed to instigate non protective antibodies that derive pathologic ADE. Early research on COVID-19 vaccines revealed that subunit vaccines that can elicit S-specific NAbs, particularly against S stabilized in the prefusion conformation, diminish the presentation of non-neutralizing epitopes and are hence associated with lower ADE risks (73). A recent study by Wang et al., however, has indicated that the full-length S protein of SARS-CoV-2 used for vaccine development induces nNAbs that increase the rate of ADE of infection by facilitating virus entrance into host cells that possess complement or Fc receptors and exacerbating the viral infection (178). This suggests that approaches for developing vaccines should lessen any potential immunopathology linked to nNAbs (125).

On the other hand, COVID-19 immunization strategies that use the RBD of the S protein have been demonstrated to stimulate high titers of NAbs and long-lasting humoral protective immunity, providing a high chance of success with minimal risk of ADE (179, 180). This highlights that vaccines containing RBD directly or vectors encoding the RBD sequence may be considered an ideal, safe vaccine for protection against SARS-CoV-2 infection. This is in agreement with other studies showing that replacing the full-length S protein with RBD as the antigen lowers the risk of ADE while improving the immunogenicity of the candidate vaccine (125, 181). However, opposing results have been reported by Walsh and colleagues, who compared two mRNA-based COVID-19 vaccine and showed that the BNT162b2 vaccine, encoding the entire length-S protein was associated with a lower incidence and severity of systemic reactions than the BNT162b1encoding the RBD of the S protein (182). These counterintuitive results support the necessity for additional research to pinpoint the best potential viral antigen for designing safer COVID-19 vaccines.

Furthermore, a pathological type 2 inflammatory response, which is accompanied by a eosinophilic pulmonary immune pathology, could also be a concern during COVID-19 vaccination, as reported by some studies (25–27). Nonetheless, several other preclinical studies of COVID-19 vaccines found no evidence of increased respiratory disease typical of an increased eosinophilic proinflammatory pulmonary response upon challenge in preclinical studies of COVID-19 vaccines (27, 183). But the risk of Th-2-oriented immune serum following COVID-19 vaccination that contains polyclonal antibodies directing the S protein to boost virus infection needs to be further evaluated by taking into account the stated risk of ADE of infection. To improve the efficacies and safety of COVID-19 vaccines, careful selection of antigens during vaccine development to induce high-affinity NAbs polarizing the T-cell response towards type 1 immunity while suppressing unwanted type 2 immunity may be critical (26). In addition, adopting adjuvants that promote Th1 response polarization, reduce the risk of undesired type 2 immune response, and hence improve the protective effect of COVID-19 vaccines have been shown to have promising outcomes (25).

Even though all currently authorized COVID-19 vaccines are ideally safe, effective, and less risky of developing severe COVID-19 illness, there are some associated mild symptoms such as pain, redness, swelling, tiredness, headache, muscle pain, chills, fever, and nausea after vaccination (184). Rarely, some severe and even fatal symptoms such as blood clotting may happen following the administration of a few vaccines. For instance, AstraZeneca and JNJ-78436735 vaccines are recently reported to be associated with very rare cases of blood clotting with low blood platelets (thrombocytopenia) with or without bleeding, including rare cases of clots in the vessels draining blood from the brain, particularly in women younger than 50 years old. But many countries continue to use the vaccines since this fatal type of side effect has an extremely rare occurrence and the benefits of the vaccines outweigh the risk of side effects (185–188). Moreover, Guillain-Barre Syndrome was reported following JNJ-78436735 and Pfizer COVID-19 vaccination (189, 190).

The other challenge associated with compromised vaccine efficacy is the emergence of new SARS-CoV-2 variants. So far, numerous SARS-CoV-2 variants have been reported to appear and spread globally, including B.1.1.7, B.1.351, P.1, P.2, B.1.427, B.1.429, B.1.525, B.1.526, B.1.617, B.1.1.298, B.1.1.207, and B.1.1.529 (191, 192). Naturally occurring mutations in the new SARS-CoV-2 variants span the whole length of S protein but mainly occur in S1 and RBD which are the main targets of NAbs and may alter their interaction with the ACE2 receptor (156, 193). The main RBD mutations of concern, namely N501Y, E484K, K417N, and K417T, have been identified as antigenically important and reported as escape mutations for several mAbs. The B.1.1.7, P.1, and B.1.351 variants have the N501Y and E484K substitution in S protein when compared to the WIV04/2019 (or the reference or original) sequence. In addition, a K417T substitution is present in both B.1.351 and P1 lineages, and K417N mutation occurs in the B.1.351 variant (193–195). The current suite of antibody vaccines was designed with S protein based on strains circulating during the early phases of the pandemic. This raised a concern as to whether the currently approved vaccines will be effective against the new variants of SARS-CoV-2. Thus, the emerging strains of SARS-CoV-2 could pose another challenge to developing an effective COVID-19 vaccine, as has happened with other viral vaccines (193–195).

According to available data, antibodies developed in response to the original SARS-CoV-2 infection or vaccines found to provide protection (neutralization) against some strains, such as D614G, and antibodies developed in the majority of patients can cross-neutralize D614 or G614 strains, lowering the risk of re-infection (9, 130, 196).In contrast, preliminary studies with pseudo viruses suggest that neutralization by most convalescent sera and vaccine-induced immune sera is largely ineffective and has limited cross-protection against the recently emerged novel SARS-CoV-2 variants (193–195). Some of the novel SARS-CoV-2 variants that have particular importance, known as variants of concern (VOC), generally increase the transmission dynamics, virulence, and disease severity as well as significantly suppress the host immune response, such as neutralization by antibodies produced during prior infection or vaccination, decreasing therapy or vaccine effectiveness against them, or causing diagnostic detection failure (197). **V**ariants with multiple substitutions in S protein, including in the NTD and RBD, enhance transmissibility, which may explain why SARS-CoV-2 transmission is still uncontrollable and the world is in the midst of a COVID-19 pandemic. Besides, the novel SARS-CoV-2 variants containing K417N, K417T, E484K, and N501Y RBD mutations in the S protein gene, namely B.1.1.7, B.1.351, and P.1 have been shown to reduce the overall neutralization efficacy of the current COVID-19 vaccines, such as AZD1222, JNJ-78436735, NVX-CoV2373, and Coronavac (153, 154, 193, 198).

Generally, these variants may significantly reduce neutralization, even in fully vaccinated individuals (199–201). This could be due to the higher immune evasion potential of SARS-CoV-2 amplifying the waning of humoral immunity produced in response to vaccination. Therefore, the reformulation of existing vaccines to incorporate diverse spike sequences should be considered during vaccine development to produce new vaccines capable of eliciting broadly neutralizing antibodies (bNAbs) that may be necessary to resolve the ongoing pandemic as well as prevent future pandemics from novel strains. Other studies have suggested that waning immunity, rather than immunological escape, may be to blame for the gradual decline in vaccine effectiveness (202, 203). This suggests that a booster dose of the COVID-19 vaccine appeared to increase neutralizing activity and confer prolonged protective immunity against SARS-CoV-2 variants. Mounting body of evidence shows that a booster dose of the mRNA vaccine appears to increase neutralizing activity against the omicron variant, reducing symptomatic disease and/or hospitalization; although protection against symptomatic omicron infection is less effective than with Delta, and the immunity may fade over time (204–207). Therefore, additional booster doses (two or above doses) may reestablish antibody levels and increase effectiveness against all COVID-19-related outcomes; however, the protection against infection remains short-lived (208, 209).

## 6 Conclusion

In summary, NAbs are components of the humoral immune response produced against the S protein of SARS-CoV-2, particularly the RBD of the S1 subunit that has a key role in viral entry and infection of target cells. NAbs bound to RBD block the SARS-CoV-2 from invading the host cells and its replication with or with no other immune cell involvement. This process of viral clearance by disabling its infectivity is called neutralization. Potent NAbs are essential to protect the body from SARS-CoV-2 infection or reinfection. NAbs have also potential application in clinical settings and can be generally helpful in the diagnosis of COVID-19, determination of immune status and duration of protection against SARS-CoV-2, as well as assessment of disease severity, clinical outcome, and vaccine effectiveness of COVID-19. Moreover, NAbs have been reported to play a therapeutic and prophylactic role in COVID-19 if the antibodies have high potency and are safe with a low risk of developing adverse events. They are also crucial in COVID-19 vaccine development, with several of them having received approval currently in many countries. Despite all these implications, COVID-19 antibody-based prophylaxis, therapy, and vaccination are possibly associated with unwanted immune responses such as eosinophilic type 2 immune response and ADE. In addition, the newly emerged variants of SARS-CoV-2 may reduce vaccine efficacy and challenges in developing an effective COVID-19 vaccine. Thus, producing new vaccines by incorporating diverse spike sequences to elicit bNAbs may be needed.

## Author contributions

The first draft was designed by EC and both EC and TA made a significant contribution to the work reported, whether that is in the compilation, editing, and finalizing the review. All authors contributed to the article and approved the submitted version.
